# Dr. Abercrombie on the Pathology of the Stomach

**Published:** 1830-07-01

**Authors:** 


### XXVI.

### Pathology of the Stomach and Intestinal Canal, &c. By J. Abercrombie. M. D.*

We have already noticed two or three of the additional cases inserted by Dr. Abercrombie in the last edition of his valuable work, and we have now to throw a coup d'œil on the remainder. They are few and by no means important, being rather designed to fill up hiatus in the morbid anatomy of the organs treated of, than to afford matter for practical reflections. We will take them as they come.

Case I. (lv) *Fatal Peritonitis after Drinking Cold Beer, during Profuse Perspiration.*

A man, æt. 60, whilst perspiring profusely, on the 22d of June, 1829, drank two quarts of cold beer. During the following night he was attacked with

* Second Edition, enlarged, 1830.

severe pain and sudden distention of the abdomen, with noise in the right hypochondrium; vomiting was added on the 23d; no passage through the bowels could be procured, though enemata brought away bloody stools; and on the 26th he was admitted into the Edinburgh Infirmary with the symptoms of peritonitis. Every thing which Dr. Duncan could do was done, but the patient sank on the following evening.

*Sectio Cadaveris.* "The small intestines were much distended and were filled with a fluid of a yellow colour, similar to that which had been vomited. They were externally much injected, with some adhesions. In their substance they were easily torn, giving way even when gently handled. The lower end of the ileum and the caput coli were of a deep red or port-wine colour. The great intestines contained chiefly gas, and a small quantity of fluid feces, and no appearance was discovered of any contraction or obstruction, except what arose from a slight narrowing of the ileum near the ileo-colic valve. At this place there existed an ulcer, which extended quite round the circumference of its inner surface, and was about an inch in breadth. It had gangrenous edges, and the bottom of it seemed to be bounded only by the peritoneum, the mucous and muscular coats being destroyed. The man had enjoyed perfect health up to the period of this attack."

It is most probable that the inflammation of the peritoneum in this instance was consecutive to that of the mucous membrane, in the vicinity of the ileo-cæcal valve. This is what we frequently observe in the progress of continued fever, and is most consistent with the nature of the exciting cause of the disease, the application of cold to the mucous surface of the stomach and intestines. Dr. Abercrombie cites it to bolster up his unfortunate theory of ileus, but we will only say that his remark, "the case can only be explained by the supposition of sudden distention and loss of muscular power," is purely hypothetical, and altogether unsupported by, indeed incapable of, proof.

CASE 2. (CXII.) :—*Fatal Peritonitis from Perforation of the Intestine in Fever.* A boy, aged 10, in February 1829, was affected with the mildest form of the epidemic fever at that time prevalent in Edinburgh. His pulse was scarcely 100; his bowels were easily regulated, and the motions quite healthy; and the abdomen was entirely free from pain, tension, or tenderness. In this favourable state of all the symptoms, he went on to the 12th day. He was then suddenly seized with most intense pain of the abdomen, with vomiting; the abdomen soon became tense, tender, and tympanitic; the pulse was rapid and feeble. I now saw him for the first time, along with Dr. Robert Hamilton. No relief was obtained from any kind of treatment; he continued in a state of extreme and continued suffering, and died in about 30 hours.

*Inspection.*—The peritoneal cavity was distended with air, and contained some liquid feces. There were the usual appearances of extensive but recent peritonitis. In the lower extremity of the ileum there were five or six small but well-defined ulcers, no larger than the diameter of a split-pea, one of which had perforated the intestine by a round aperture. The seat of these ulcers appeared to be in the mucous follicles, and the disease from which they arose was distinctly traced at different spots, in different periods of its progress; namely, first a firm, elevated nodule or tubercle, then a pustule, and then an ulcer.

The foregoing is a good specimen of a class of cases with which the profession are now becoming conversant. To say that they are insidious would be merely to echo a truism, but the fact is, that in some cases the symptoms are more than obscure, they are absolutely incapable of being unravelled till the sudden and terrible onset of coffee-ground vomit and abdominal pain proclaim that perforation of the gut has taken place, and that the patient's death warrant is sealed. In more than one instance we have seen the thoracic symptoms so prominent as almost to forbid suspicion with respect to the abdomen, and yet the foregoing train of symptoms have set in, the patient has rapidly sunk, and no more than the usual congestion of fever has been discovered in the lungs. Practitioners will do well to bear these facts in mind, but they should also be aware that all or nearly all the symptoms of implication of the intestinal mucous membrane may exist in fever, and yet on dissection that membrane will be found to be next to healthy. We lately saw a case of this kind, in which the black tongue, stinking excretions, and other features of the case gave every reason to believe that the intestines were affected. On dissection some slight congestions and nothing more were found in the colon, but the lungs were crowded with myriads of miliary tubercles. It is curious that the lungs and the intestines should thus play into each other's hands in the progress of fever.

Case 2. (cxxi.) :—*Disease of the Omentum.* "A lady, aged 60, of a full habit, had complained for some months of prominence, weight, and habitual uneasiness in the front of the abdomen. In November 1823, the complaint assumed an acute character, with severe pain, affected by respiration, and fever, but without obstruction of the bowels. The pain was increased by pressure, and a soft diffused tumour was felt to occupy the epigastric and umbilical regions, without any distention of the abdomen. The usual antiphlogistic treatment was now adopted, but with only partial and temporary benefit. After two or three weeks, the pain had become much less urgent; but she then passed into a state of low fever, with occasional delirium, and she died at the end of five weeks from the commencement of the acute attack. For the last week of her life, there was retention of urine, requiring the use of the catheter.

"*Inspection.*—The disease was found to be entirely in the omentum, which formed a thick, fleshy mass between three and four pounds in weight. It was of a dark colour and soft consistence, and no disease was detected in any other organ."

This case was communicated to our author by Dr. Storer, of Nottingham, and is an instance of what is but rarely seen, uncombined disease of the omentum. Dr. A. observes that Dr. Strambio has described another form of omental disease in the Annali di Medicina. It formed an immense cerebral tumour, involving the spleen, the left kidney, the ovaria, uterus, and rectum; the symptoms were vomiting with enlargement of the abdomen and febrile paroxysms. Such cases as these are not so rare, for we have seen, and most of our brethren must have done the same, several instances of malignant disease involving the omentum with other parts. It is disease of the omentum solely which is uncommon.

Case 4. (cxxiii.) :—*Very obscure and peculiar Affection, with Symptoms chiefly referable to the Bowels.* This occupies a short section of our author's work, and is not undeserving of attention. Dr. Abercrombie remarks that the affection would appear to be connected with some morbid condition of the mucous membrane of the intestinal canal, the precise nature of which eludes our observation. The patient is thin, pale, weak, with a withered look, a peculiar dry state of skin, and a small weak pulse. His appetite is variable and capricious, and he feels uncomfortable after eating; the bowels are slow, though easily regulated; and the evacuations are always of a remarkably dull colour like mahogany, or almost black. The following fatal case will illustrate the foregoing description.

"A lady, aged about 30, had been in bad health for four or five months; and when I saw her, was wasted like a person in an advanced stage of phthisis. She had a small frequent pulse and bad appetite, but complained of nothing except some undefined uneasiness in the abdomen. The bowels were slow, requiring the constant use of medicine; the motions were consistent and formed, but always of the deep brown colour of dark mahogany or rose wood, and no treatment had any effect in correcting that colour. The abdomen was collapsed, and nothing could be discovered by examination. Sometime after I saw her, she began to have uneasiness in her chest, with slight cough; she then became liable to fits of coma, in which she lay with her eyes open, but unconscious of any thing; at length she had repeated paroxysms of convulsion, and she died in a state of the most extreme emaciation, after an illness of eight or nine months duration.

"*Inspection.*—No disease could be discovered in the brain, and the lungs were quite healthy, except some very old adhesions of the pleura. The intestinal canal was throughout so thin, as to be transparent like goldbeater's leaf. On the mucous membrane there was in many places a tenacious mucus of a dark brown colour, but no disease could be discovered in the membrane itself, and no morbid appearance could be detected in any organ."

Dr. A. does not attempt to explain the case, indeed the only conjecture he can offer respecting it is, that some morbid condition of the mucous membrane interfered with digestion, and prevented the nourishment of the body. Whether this be or be not the fact, we are sure that we have seen others of no very dissimilar character. We remember, for instance, a case of chronic dysentery or rather diarrhœa, for the motions were never tinged with blood, which obstinately resisted every kind of remedy, and on dissection no disease of the least consequence could be discovered in the body. In another instance of vomiting, fetid evacuations, and extreme emaciation, dissection after death revealed no morbid appearances of any moment. Those who have seen much of morbid anatomy will acknowledge that such cases are not of the rarest, and they offer a humiliating commentary on the imperfection of our art, as well as on the extravagant fanaticism of the ultra apostles, of what we may term sectio-cadaverism.

"I have seen some other cases which showed similar characters, and proved very tedious and unmanageable. The peculiar character in all of them was the remarkably dark colour of the evacuations, which nothing had any effect in correcting. The last case that occurred to me seemed to derive most benefit from the sulphate of iron; and this remedy, which in general makes the evacuations very dark or nearly black, made them in this case decidedly lighter than their usual colour. Another seemed to derive benefit from small quantities of mercury. The patients had in general a peculiar emaciated withered aspect, with a dry state of the skin, a weak pulse, and a variable and capricious appetite; but no actual disease could be discovered capable of accounting for their unhealthy appearance."

CASE 5. (CXXVI.):—*Ileo-vesical Communication, from the Metastasis of Rheumatic Inflammation.* A lady, æt. 63, was seized, June 1829, with rheumatic symptoms accompanied by an erythematic blush on the ankles. In eight or ten days these symptoms disappeared rather suddenly, and dysuria with much uneasiness in the region of the bladder supervened. Next day, July 9th, there was complete retention of urine, pain and distention of the abdomen, continued vomiting, rapid feeble pulse, and cold skin. On the 10th the vomiting subsided, but the retention continued, and much bloody urine was regularly drawn off by the catheter. This state of the urine ceased in eight or ten days, and it then became highly offensive, depositing pus and slough, and on several occasions a quantity of fetid gas escaped through the catheter. The abdomen continued distended, the motions were liquid and offensive, and on the 28th she died.

*Sectio Cadaveris.* "The omentum adhered to the bladder and to the ascending colon. The caput coli was greatly enlarged, and the extremity of the ileum adhered to the posterior part of the bladder. The bladder adhered extensively to all the parts within the pelvis, and in attempting to separate it, a large quantity of pus escaped. Its inner surface was sloughy, and shreds of its mucous coat were hanging into its cavity. An opening capable of transmitting a goose quill was found to exist betwixt the bladder and the portion of ileum which adhered to it. The left kidney was healthy; the right was wasted, so as to leave only the calyces and cellular texture without any of the glandular structure."

CASE 6. (CXLII.):—*Fungus Hæmatodes of the Liver ulcerating into the Stomach.* A woman, æt. 30, was affected with cough, copious expectoration of viscid mucus, night-sweats, and great prostration of strength. In Nov. 1824, she was seized with vomiting of dark matter resembling venous blood partially decomposed, and she discharged large quantities of the same by stool. A hard moveable tumour was felt in the epigastrium, which was painful on pressure. Her strength now sank rapidly, and she died on the 3d of December, the vomiting having ceased several days before her death, but the cough continuing severe, the bowels being obstinate, and the motions as before.

*Sectio Cadaveris.* "The tumour that had been felt in the epigastrium was found to be a tubercle, the size of an egg, attached to the left lobe of the liver. It adhered firmly to the stomach, near the pylorus; and on the internal surface of the stomach, at the place of the adhesion, there was an ulcer the size of a shilling; this ulcer appeared to have been the source of the black discharge, a considerable quantity of which was still found in the stomach and intestines. The coats of the stomach, along nearly the whole of the smaller arch, were much thickened and indurated, and the pylorus was considerably contracted in its aperture. The tubercle presented, when cut into, a variegated texture, partly a firm white tubercular matter, and partly a reddish substance resembling the structure of the liver; but the white matter was the more abundant. There were four or five similar tumours, the size of walnuts, in various parts of the liver. The left extremity of the pancreas was of a soft cheesy consistence, and adhered to the stomach. The other abdominal viscera were healthy. After the most careful examination, no disease could be discovered in the viscera of the thorax, except a few slight adhesions between the pleura costalis and pulmonalis, which were evidently of long standing."

The above is related as an instance of "tubercular disease" of the liver; it was evidently fungus hæmatodes. We lately saw a case of fungus hæmatodes of the stomach in which the coats of the organ at the pyloric extremity and along the lesser curvature were perforated by ulceration, whilst the cavity of the stomach communicated with that of an abscess situated between it and the liver. In this case the symptoms were so obscure as scarcely to direct suspicion to the affected organ.

Case 7. (cl.):—*Fatal Hæmorrhage from the Spleen.* "A woman, aged 20, was admitted into the Infirmary of Edinburgh, on 16th June, 1829, under the care of Dr. Duncan. Her complaints were chiefly of a rheumatic character, with considerable nausea, some fever, anxiety, and restlessness. She stated, that, a fortnight before, she had been suddenly seized with severe pain in the stomach, followed by nausea and vomiting, and that these symptoms continued to recur at intervals for a week. On the 17th, there was vomiting, with much anxiety and restlessness, and she complained of pain on pressure in the left side beneath the false ribs. On the 18th, she became low and cold, and died in the evening.

"*Inspection.* A quantity of coagulated blood was found in the cavity of the abdomen, which was ascertained to have proceeded from a laceration of the spleen. That organ was of a paler colour than natural, and its substance was soft and easi y torn. There was a sacculated disease of the right ovarium; but no other appearance of recent disease could be detected in any organ."

Such cases as the foregoing are uncommon but not unique. Dr. Abercrombie refers to one mentioned by Fournier, in which a man who had suffered for some months from quartan ague died suddenly, when convalescent, after a hearty supper. The spleen was found enlarged and ruptured, and there was much coagulated blood in the cavity of the abdomen. Within this last fortnight we have heard of a similar case in this metropolis. The patient died after a short illness, and the cause was supposed to be internal hæmorrhage of some kind or other. On dissection the spleen was found to have been ruptured and the blood to have escaped into the peritoneal cavity. Of the history of the case we know nothing, as we only heard of it indirectly.

Case 8. (cli.):—*Enlargement and mixed State of Disease of the Pancreas.*

"A lady, aged about 40, came to Edinburgh in May, 1829, affected with very deep jaundice, which was of several months standing. There was occasional uneasiness in the abdomen, but it was not severe; and the general health was little impaired. No disease could be discovered in the region of the liver: in the centre of the abdomen, near the umbilicus, there was a slight feeling of knotty irregularity, but it was obscure, and could only be felt occasionally. I saw her along with Dr. Macwhirter, and a great variety of treatment was adopted without benefit. She at length became dropsical, and returned to the country, where she died, gradually exhausted, in August.—I am indebted to Mr. Syme of Kilmarnock, for the account of the morbid appearances.

"*Inspection.* There was a gallon of fluid in the abdominal cavity. The gall bladder was very large, and was distended with very black bile. The liver was of a deeper colour than natural, but otherwise sound. The whole of the peritonæum was somewhat thickened. The pancreas was enlarged to the size of two fists, and embraced the ductus communis so firmly, that it was found impossible to pass a probe from the gall bladder into the intestine. It was of a mixed texture, some portions being soft, resembling the medullary sarcoma, and others of scirrhous hardness. The other viscera were healthy."

This completes the set of cases appended to the last edition of Dr. Abercrombie's work, and concludes likewise our brief sketch. We have already expressed a very favourable opinion of the volume, and it would almost be superfluous to say, that we recommend it to all branches, and each member of the profession.

---

